# Editorial: Optogenetic Tools in the Molecular Spotlight

**DOI:** 10.3389/fmolb.2016.00014

**Published:** 2016-04-28

**Authors:** Tilo Mathes, John T. M. Kennis

**Affiliations:** Department of Physics and Astronomy, Vrije Universiteit AmsterdamAmsterdam, Netherlands

**Keywords:** photoreceptors, optogenetics, flavins, opsins, phytochrome, spectroscopy, computational modeling, protein engineering

Photosensory receptors have been in the center of vision research and photobiology since the discovery of rhodopsin in 1876 by Franz Boll. However, in the last 10 years the rise of optogenetics has placed them in a broader focus. The majority of these biological light-sensors consist of a protein/pigment complex that alters the activity of a cognate biological effector upon absorption of a photon. With the nowadays available information on the corresponding genes, proteins and the vast access to (meta-) genomic data as well as sophisticated methods in molecular biology and genome engineering the photoreceptor principle of transforming light into biological information is now exploited in many different fields of research.

Photosensory receptors therefore not only constitute the backbone of a major methodological breakthrough in cell and neurobiology but also offer bright perspectives for our understanding of dynamic biomolecular processes in general. The possibility to use photons as substrates enables researchers to induce and experimentally monitor biomolecular reactions with up to femtosecond resolution. Combined with techniques capable of molecular resolution such time-resolved experiments not only provide dynamic molecular information on the underlying mechanisms of photosensory and general signal transduction, but also will enable us to identify structure/function relations and design principles of biological sensor/effector complexes. Ultimately, this knowledge will allow us to rationally design novel light-responsive tools with customized properties for application in optogenetics and synthetic biology.

This ebook features research articles and reviews covering the most prominent photosensory modules applied in optogenetics, as represented by flavin based photoreceptors (LOV and BLUF), phytochromes and microbial opsins. The articles of this collection showcase state-of-the-art approaches to elucidate the molecular function of such photosensory modules from the initial event of photon absorption to the activation of a downstream effector.

Ritter et al. summarize recent advances in time resolved infrared absorption spectroscopy, which allows researchers to visualize structural changes involved in the activation of photosensory proteins by identifying changes in vibrational frequencies of individual chemical bonds. While infrared spectroscopic data may therefore become extremely complex in proteins, larger scale structural transformations like domain rearrangements in photosensor/effector complexes and their dynamics can be more efficiently mapped by discrete distance measurements between interacting paramagnetic centers using pulsed electron paramagnetic spectroscopy as illustrated by Nohr et al. Another elegant way of characterizing functionally relevant differences in signaling-active and inactive protein forms is presented by Lindner et al. Their review summarizes hydrogen/deuterium exchange mass spectrometry that provides information on solvent accessibility and domain flexibility, and thus important mechanistic insights on how protein dynamics determine signal transduction.

In their research article, Song and coworkers employ magic angle spinning solid state nuclear magnetic resonance spectroscopy to investigate the molecular and electronic structure of the protein-embedded tetrapyrrole cofactor (Song et al.). The chromophore and its dynamic interaction with the protein environment can be studied with extremely high molecular resolution using this technique and allowed the authors to determine aggregation and hydration effects induced by sample preparation on the local structure of the chromophore. Such insights are crucial to critically evaluate experimental results and their functional implications.

Lehtivuori et al. also investigate the molecular environment of the phytochrome chromophore and combine various methods to identify structural prerequisites and potential design guidelines for the fluorescence lifetime in phytochrome based infrared fluorescent proteins. Besides being used as optogenetic actuators phytochromes turned out to be highly attractive tools for deep tissue imaging due to the relatively high penetration of long wavelength light in tissue. Fluorescence spectroscopy and X-ray crystallography demonstrate that phytochrome fluorescence is strongly influenced by bulky residues proximal to the chromophore and by presence of water in the vicinity of the chromophore.

As photoinduced signal transduction is ultimately determined by the primary events following photoexcitation and their quantum efficiencies, ultrafast techniques with femtosecond resolution are essential. Ihalainen et al. summarize and compare the primary photochemistry of the red light absorbing phytochromes obtained by ultrafast absorption spectroscopy up to several nanoseconds. They show that the excited state dynamics are strongly affected by the length and subunit composition of the investigated proteins and suggest feedback mechanisms from the distal domains to the chromophore binding pocket, which are most likely of functional relevance.

Stensitzki et al. employ ultrafast time resolved visible absorption spectroscopy to investigate the photoactivation of the light-activated ion channel channelrhodopsin-1, a close relative of the most prominent optogenetic tool: channelrhodopsin-2. They identify a distinct ground state heterogeneity illustrated by a strong excitation wavelength dependence of the observed photodynamics that has not been observed for the well-studied channelrhodopsin-2 and discuss its implications for the activation of the protein.

Heterogeneity in receptor conformation is a recurring topic both in photoreceptor and signal transduction research and is crucial to understand dark noise of receptor proteins. Chromophore heterogenetity and its relation to signaling is also the focus of the study of Velazquez Escobar et al. on phytochromes (Velazquez Escobar et al.). They identify two far-red absorbing states in the cyanobacterial phytochrome Cph1 using a combination of steady state Raman spectroscopy, ultrafast time resolved infrared spectroscopy and quantum chemical calculations.

In addition to experimental methods as described in the articles above, computational methods are powerful approaches to calculate spectroscopic properties or molecular dynamics under selected conditions, which may not be accessible experimentally. In this collection computational methods are used to support experimental findings by calculating vibrational frequencies of chromophores (Velazquez Escobar et al.) and to simulate molecular structural dynamics of photoreceptor proteins. Bocola et al. employ molecular dynamics simulations to investigate light-induced structural changes in dimeric LOV domains and provide a novel mechanism for the photoactivation of dimeric LOV photoreceptors. Mathes and Götze review the currently available computational studies on the spectroscopic properties and vibrational frequencies of BLUF photoreceptors and explore an alternative mechanism of BLUF photoactivation using quantum chemical calculations.

Finally, the ebook contains concise reviews on opsin based optogenetic tools and modular photoreceptors that illustrate how the molecular insights that we obtained so far can be applied to rationally design novel photoswitches with customized activities. Ziegler and Möglich provide a thorough overview on modular photoreceptor function, architecture and design principles. The review of Pudasani et al. focuses on the structural prerequisites for tuning the LOV domain chemistry and signal transduction to ultimately allow for improved LOV-domain based optogenetic tools. Kandori summarizes structure/function relations in the extremely versatile microbial rhodopsin pumps that have been proven to be key optogenetic tools.

This ebook thus provides an exciting collection of various molecular approaches to elucidate the photochemistry and signal generation in a variety of photoreceptors from absorption of a photon to the biological output that will provide researchers with fundamental knowledge to create and customize novel optogenetic tools.

## Author contributions

All authors listed, have made substantial, direct and intellectual contribution to the work, and approved it for publication.

### Funding

JK and TM were supported by the Chemical Sciences Council of the Netherlands Organization for Scientific Research (NWO-CW) through a VICI grant to JK.

#### Conflict of interest statement

The authors declare that the research was conducted in the absence of any commercial or financial relationships that could be construed as a potential conflict of interest.

